# Current Trends in AI and Eye Disease Diagnostics

**DOI:** 10.1007/s44402-026-00193-2

**Published:** 2026-09-28

**Authors:** Maria Jessica Cruz, Siddharth Limaye, Mark Christopher

**Affiliations:** 1 University of California, Davis, School of Medicine, Sacramento, California USA; 2 Viterbi Family Department of Ophthalmology, Shiley Eye Institute, University of California, San Diego, La Jolla, California USA; 3 Carle Illinois College of Medicine, University of Illinois at Urbana-Champaign, Urbana, Illinois USA

**Keywords:** Artificial intelligence, Deep learning, Digital innovations, Machine learning, Ophthalmology diagnostics

## Abstract

**Purpose:**

Artificial intelligence (AI) has rapidly advanced as an approach for ophthalmic disease detection, driven by the widespread use of high-dimensional imaging modalities (e.g., optical coherence tomography). This review summarises the machine learning and AI approaches for disease detection in ophthalmology and discusses emerging paradigms and highlights key challenges impacting clinical translation.

**Recent Findings:**

AI-based systems have demonstrated suitably high diagnostic performance across major ophthalmic diseases, including diabetic retinopathy (DR), diabetic macular oedema, glaucoma, age-related macular degeneration and retinopathy of prematurity. Several tools have even received regulatory approval for commercial DR screening. More recently, foundation models trained using self-supervised learning have enabled more generalisable and data-efficient disease detection across datasets and imaging modalities. In parallel, multimodal large language model systems are emerging that integrate imaging and clinical data to support more comprehensive diagnostic workflows. Early agentic AI systems extend this paradigm further by coordinating multiple models to perform disease detection, provide clinical decision support and generate reports.

**Summary:**

AI-based disease detection in ophthalmology has achieved substantial technical progress but only limited translation into routine clinical practice. Key barriers include technical, clinical, ethical, economic and regulatory concerns. Future efforts should prioritise prospective evaluation in real-world settings, addressing model transparency and bias and alignment with clinical and regulatory requirements. With continued advances, AI has the potential to expand access to care, improve diagnostic accuracy and reduce the global burden of vision loss.

Key points

- Artificial intelligence tools have achieved high-performance in disease detection, with systems achieving expert-level accuracy across major conditions, including diabetic retinopathy, diabetic macular oedema, glaucoma, age-related macular degeneration and retinopathy of prematurity.
- The field is incorporating newer foundation models and large language model-based tools to improve generalisability, provide additional clinical decision support and integrate with existing workflows.
- Clinical translation remains limited and addressing economic, ethical and regulatory barriers is critical to adoption.

## Introduction

Artificial Intelligence (AI) has emerged as a transformative force in medicine, with ophthalmology positioned near the forefront of pre-clinical AI research [1, 2]. The widespread use of various high-dimensional modalities such as fundus photography, optical coherence tomography (OCT)/OCT angiography (OCT-A) and visual field (VF) testing, as well as ophthalmic and systemic clinical data, creates the rich datasets critical for AI applications [3, 4]. The global burden of eye disease is large, with more than a billion people worldwide estimated to suffer from some form of visual impairment [5]. Aging populations, increasing disease rates and limited resources (especially in underserved areas) mean that the burden of eye disease will continue to increase and require new solutions to address. This makes the adoption of AI systems especially attractive. They hold the potential to expand access to care, reduce costs and preserve vision, especially in resource-limited settings [6]. However, development, validation and deployment of these systems present their own set of challenges. This review provides an overview of AI in ophthalmology, summarising the evolution of AI approaches in ophthalmology, current clinical applications and the challenges and future directions in this developing field.

## AI and Machine Learning

AI is a broad term that refers to computational systems that can perform tasks that have traditionally required human intelligence. Contemporary AI tools are built using machine learning (ML) techniques, a subfield in which systems learn patterns in data rather than relying on explicitly programmed rules [7]. ML techniques have been applied in ophthalmology for decades to support diagnosis, prediction and image analysis. ML approaches can range from traditional techniques that rely on *hand-crafted* features designed using domain expertise to the deep learning (DL) models that underpin modern generative AI (Table 1). Typically, these approaches rely on training and independent testing datasets and are broadly categorised as supervised (learning from labelled data) or unsupervised (learning from unlabelled data). Regardless of the specific methods, successful model development also requires selecting an appropriate model type and parameters to help balance bias (associated with model *underfitting*) and variance (associated with model *overfitting*) to help ensure accurate, generalisable predictions. Over the past few decades, ophthalmic researchers have applied a variety of ML techniques to fit the specific type of predictions, available datasets and performance requirements needed for their task.

**Table 1:** Common AI and ML paradigms used in ophthalmology.

Paradigm	Description	Approach	Examples	Strengths/Weaknesses
Traditional machine learning	Machine learning approaches relying on “hand-crafted” features derived from ophthalmic data and domain expertise	Features are manually defined and extracted for input into statistical learning models	Logistic regression, support vector machines [129], random forests [130] and gradient boosting	*Strengths*: Interpretable models; works on smaller datasets *Weaknesses*: Manually defined features; limited performance
Deep learning	End-to-end learning from raw data (e.g., images) to automatically identify usual features	CNNs trained on labelled image datasets for classification or segmentation	ResNet [9, 131], U-Net [132], vision transformers [133]	*Strengths*: High accuracy on image-focused tasks *Weaknesses*: Large, labelled datasets needed; limited generalisability
Foundation models	Large-scale pretrained models enabling generalisable representations	Self-supervised pretraining and fine-tuning or few-shot adaptation	RETFound [17], DINO [14], masked autoencoders [134]	*Strengths*: Generalises well to new data; self-supervision does not require labelled data *Weaknesses*: Computationally intensive; requires very large unlabelled data for initial training
LLMs/Agentic AI	LLMs make predictions, provide clinical decision support or documentation and interact with other models/tools	General-purpose LLMs fine-tuned for medical tasks and connected to other models/tools to manage the entire workflow	Med-PaLM [16], Med-Gemini [119], EyeCLIP [120]	*Strengths*: Multimodal approach; flexible workflows. *Weaknesses*: Hallucination risk; limited validation

*AI* artificial intelligence, *ML* machine learning, *CNN* convolutional neural network, *LLM* large language model.

During the late 2010s, convolutional neural networks (CNNs) were a dominant type of DL model for image-based disease detection. This type of DL model learns features that interrogate pixels within spatially defined neighbourhoods and aggregates these hierarchically to learn an overall representation of an input image [8]. This representation can then be used to assign labels to images (e.g., healthy vs. disease). All CNNs use this general approach but differ in the specifics of how they are built, giving rise to different architectures such as ResNet [9], DenseNet [10], VGG [11] or Inception [12].

More recently, a new type of DL model has come into prominence: transformers [13]. These models can better model long-range relationships in images and more flexibly handle multimodal data. Transformers are also used as the basis of modern self-supervised foundation models that learn from unlabelled data [14]. These approaches do not require large datasets of labelled or annotated images, addressing a critical challenge in ophthalmology where dataset annotation by a specialist can be costly and time-consuming. Using the self-supervised approach, foundation models are *pre-trained* on large, unlabelled datasets to produce generalisable models that can be *fine-tuned* (i.e., undergo further training on smaller, labelled datasets) to perform specific tasks such as disease detection. Different foundation models pre-trained on general-purpose (Llama [15]), medical (Med-PaLM [16]) and ophthalmic (RETFound [17], VisionFM [18]) datasets have been fine-tuned for a number of different diagnostic tasks in ophthalmology.

## Current Applications in Ophthalmology

Over the past decade, ophthalmology has emerged as an early adopter of AI and DL because it is well-suited to the strengths of these techniques. Specifically, its reliance on standardised imaging data, the well-characterised relationships between structure (measured through image) and clinical outcomes, the availability of labelled, public datasets and early regulatory successes have helped increase the speed of AI adoption in ophthalmology [2, 3].

AI systems that detect disease based on ophthalmic imaging have demonstrated robust performance for diabetic retinopathy (DR), diabetic macular oedema (DME), glaucoma, age-related macular degeneration (AMD) and retinopathy of prematurity (RoP), among other diseases. Several AI-based tools have also achieved regulatory approval for autonomous clinical deployment [19–21].

### Diabetic Retinopathy

DR screening is one of the most well-established AI-enabled screening approaches described in the literature, with real-world applications spanning web-based screening, telemedicine platforms and portable devices including smartphones [22]. Current DR classification is based on two scales: the Early Treatment of Diabetic Retinopathy Study (ETDRS) and International Clinical Diabetic Retinopathy (ICDR) [23–25]. ETDRS remains the gold standard for research and clinical trial purposes and uses a granular 15-step scale, while ICDR groups DR into five levels and is more commonly used in clinical and screening settings [26]. Most contemporary AI systems for DR detection adopt simplified, clinically applicable grading aligned with the ICDR scale [27, 28]. AI systems for DR detection demonstrate high sensitivity (88%–95%) and specificity (81%–99%) for detecting referable DR in real-world clinical settings, with performance comparable to traditional human grading [29–32]. A systematic review of 34 real-world studies found an overall pooled accuracy of 81%, sensitivity of 94%, specificity of 89% and diagnostic odds ratio of 128 [29].

In the United States, three autonomous screening systems achieved US Food and Drug Administration (FDA) authorisation for DR screening. In 2018, LumineticsCore (Digital Diagnostics, digitaldiagnostics.com, formerly IDx-DR) was the first FDA-approved model in any field of medicine, with the pivotal trial achieving 87.2% sensitivity and 90.7% specificity for detecting moderate or higher-severity DR [19]. This landmark approval established the regulatory pathway for subsequent, autonomous AI systems in the clinical space. Subsequently, EyeArt (Eyenuk, eyenuk.com) received FDA approval in 2020, with its multicentre trial demonstrating 95.5% sensitivity and 85.0% specificity for more-than-mild DR and 95.1% and 89.0% sensitivity and specificity, respectively, for vision-threatening DR [20]. Finally, AEYE-DS (AEYE Health, aeyehealth.com) was approved by the FDA in 2022, achieving sensitivity and specificity of 93% and 91.4%, respectively [33]. These systems were designed for deployment to primary care settings to be used by technicians without prior retinal imaging experience. All three were able to detect DR in patients with moderate to proliferative DR. In 2026, the Standards of Care by the American Diabetes Association recognised these systems as appropriate screening strategies with appropriate pathways for timely referrals when indicated [21]. Outside the USA, AI-based DR screening has been approved for full or pilot clinical deployment in a number of areas, including Australia, China, EU, India, Singapore, Thailand, UK and others [30, 31, 34–43].

### Diabetic Macular Oedema

Many AI/DL DR systems also screen for DME; however, these systems typically rely on the same fundus photographs used for DR detection. Since they are fundus photo-based, they have to rely on surrogate features (e.g., hard exudates), rather than detecting retinal thickening directly. Nevertheless, fundus-based algorithms achieved a pooled area under the receiver operator characteristic (AUROC) of 0.964, sensitivity of 92.6% and specificity of 91.1% in identifying DME [44]. OCT-based algorithms demonstrate somewhat higher performance, with a pooled AUROC of 0.985, sensitivity of 95.9% and a specificity of 97.9%, reflecting the OCT’s ability to visualise retinal fluid directly [44]. A multitask DL system achieved consistent performance across three common OCT devices (Zeiss Cirrus [Carl Zeiss Meditec, zeiss.com/meditec], SPECTRALIS [Heidelberg Engineering, heidelbergengineering.com], and Triton [Topcon Healthcare, topconhealthcare.com]) and seven international datasets, demonstrating its device-agnostic applicability [45]. These methods also have the advantage of providing quantitative DME biomarkers (e.g., fluid volume) [46, 47].

The three FDA-approved systems (LumineticsCore, EyeArt and AEYE-DS) are specifically authorised for detecting both more-than-mild DR and DME as separate conditions [21, 48]. Similarly, SELENA+ (Synapxe, synapxe.sg), THEIA (Toku Eyes, tokueyes.com), ARAS (Beijing Airdoc Technology Co., Ltd, airdoc.com) and several CE-marked European systems also include DME detection [28, 39, 40]. However, training on DR does not automatically confer diagnostic ability for DME. For example, AIDRScreening (Shenzen SiBright Co., Ltd.) was not trained and assessed on DME diagnosis from fundus photography, which could limit its use for comprehensive screening [38].

### Glaucoma

Glaucoma has also attracted serious DL effort, with models built to make predictions based on fundus photographs, OCT imaging, perimetry and other clinical measurements. Early landmark work demonstrated the potential of DL: Ting et al. [28] used 500,000 fundus photographs to train a multi-disease model that included glaucoma diagnosis among its capabilities. Liu et al. [49] demonstrated DL based on fundus photographs could achieve sensitivities > 96% and specificities > 97%, demonstrating performance considered comparable to specialists. Others have reported DL models with clinician-level accuracy in detecting glaucoma and glaucomatous VF defects from OCT [50–52]. DL models applied to VF data have demonstrated comparably high performance in glaucoma detection and low errors in predicting future VF summary statistics (e.g., ~1.5–2.0 dB for mean deviation) [53–55]. These modality-specific efforts reflect a broader pattern identified by Thompson et al. [56], who organised the glaucoma DL literature around three streams (fundus photography, OCT and perimetry), noting that fundus-based models carry particular appeal due to the low cost and high availability of fundus imaging, particularly in low-resource settings. Aggregate performance across these approaches has been impressive: A 2022 meta-analysis of 105 models reported an AUC of 0.98 for fundus models and 0.95 for OCT models [57] in detecting glaucoma. Another 2022 meta-analysis reported a comparable pooled AUC of 0.96 [58]. More recent work focusing on ophthalmic foundation models has shown that they achieve comparable overall glaucoma detection AUCs for individual modalities and multimodal approaches [18, 59–61]. However, there is some evidence that the foundation models are able to generalise better to new, unseen datasets and can achieve high performance even when fine-tuning data is limited to image counts less than 2000 [59, 61].

A complicating factor in glaucoma detection is the lack of a single, standardised disease definition and poor inter-rater agreement by specialists [56, 62, 63]. These issues make developing ground-truth disease labels for model training and evaluation difficult. Several approaches have been proposed to address these issues, including a “machine-to-machine” approach in which models are trained to predict some objective quantity measured using a different modality (e.g., retinal predicting layer thickness from fundus photographs) [64] and training models on longitudinal data to detect individualised change over time [65]. Despite these alternatives, there remain issues in AI/DL approaches for glaucoma, including a lack of standardisation of disease labelling as well as heterogeneity across datasets and underrepresentation of patient subgroups. Collectively, these challenges can make it difficult to compare performance across studies directly and confidently estimate model generalisability.

### Age-Related Macular Degeneration

AMD similarly emerged as an early target for AI and DL in ophthalmology. Lee et al. [66] provided one of the earliest demonstrations of CNN-based detection of AMD, classifying macular OCT images as normal or diseased. Kermany et al. [67] extended this approach using transfer learning—pretraining on a general image dataset and fine-tuning on retinal OCTs—to classify multiple conditions, including dry and neovascular AMD, achieving performance comparable to fellowship-trained specialists. The next phase in this research area shifted focus toward clinical readiness. DeFauw et al. [68] used a segmentation-first architecture in which OCT volumes were decomposed into tissue maps while a second network generated referral recommendations for multiple macular conditions, including AMD, achieving performance comparable to retinal specialists across multiple imaging devices.

Several other groups have focused not only on AMD detection but also on predicting clinically relevant disease features and treatment recommendations. Schlegl et al. [69] quantified macular fluid volume from OCT to help guide anti-VEGF treatment intervals. Bogunovic et al. [70] predicted anti-VEGF injection quantity during as-needed maintenance treatment of AMD. Gallardo et al. [71] demonstrated that the need for injection at intervals ≥ 10 weeks can be predicted from OCT, not only in AMD, but also in retinal vein occlusion and DME. Modern ophthalmic foundation models have also demonstrated that they can predict fellow eye conversion from dry to neovascular AMD within 1 year of imaging [17]. Other notable research directions include longitudinal scan series analysis for detection of structural progression [72] and a growing body of work incorporating genetic information either as model input [73, 74] or output [75], generating polygenic AMD risk scores directly from imaging data.

### Retinopathy of Prematurity

RoP is concentrated in a neonatal population where specialist access is frequently limited, making deployment of AI/DL systems for detection appealing. Brown et al. [76] provided an early demonstration of feasibility, applying a CNN to fundus imaging with ground truth established by consensus from eight expert graders. They achieved performance comparable to or better than the majority of the RoP experts on the same images. Subsequent work has also sought to address the subjectivity in grading RoP, particularly for the severe vascular variant known as Plus disease. Redd et al. [77, 78] combined DL vessel segmentation of neonatal fundus imaging with a classification model to establish the continuous Imaging and Informatics in ROP  (iROP) DL score, ranging from 1 (no disease) to 9 (severe RoP). Their goal was to operationalise what had previously been a categorical clinical judgement as a continuous spectrum. This score was later validated against the International Committee of RoP and shown to correspond with zone, stage and extent of high-grade and plus disease [79–81].

Other work has focused on real-world concerns of deploying these systems for RoP screening. Morrison et al. [82] conducted a cost-effectiveness analysis suggesting that autonomous AI screening was as effective and less costly than telemedicine and ophthalmoscopic screening. While promising, this analysis is model-based and does not include prospective evaluation of a real-world deployment. Whether AI/DL screening systems can meet the operational challenges in low-resource settings remains to be demonstrated.

## Clinical, Ethical and Regulatory Considerations

Despite the recent advances in AI/DL in ophthalmology, translation from research settings to clinical practice remains limited. Most AI systems never progress beyond research or proof-of-concept stages, with only a small number achieving regulatory approval and real-world deployment. For example, among more than 400 AI tools developed for DR, fewer than half have undergone clinical validation and only a small subset have received regulatory clearance [83]. This limitation in clearance and eventual clinical adoption contrasts with the status of AI in fields such as radiology, which has integrated computer-aided diagnosis and report generation tools [84], interventional neurology, which has implemented AI for triage and automated putative neuroimaging analysis [85] and dermatology, where implementations of AI have been used for triage in the absence of specialists and diagnosis of high-confidence cases [86]. The gap in AI system adoption between ophthalmology and other medical fields reflects a combination of technical, clinical, economic, ethical and regulatory challenges that must be addressed to enable widespread deployment.

### Clinical, Technical and Financial Considerations

One important barrier to deployment is limited generalisability. AI models trained on particular research datasets often exhibit performance degradation when applied across different populations, imaging devices or clinical environments. Importantly, model performance may vary across demographic subgroups, including race, ethnicity, age and socioeconomic status, particularly when training datasets lack diversity [87, 88]. Inconsistent reporting of demographic variables in validation studies also hinders assessment of model bias and generalisability [89]. These issues raise concerns about reliability and equity in real-world settings. Data quality further complicates clinical deployment. In routine practice, a substantial proportion of images may be ungradable, with reported rates ranging from 2.7% to 26%, potentially limiting the effectiveness of automated screening systems [88, 90]. Limited availability of large, well-annotated public datasets (particularly for OCT imaging) also makes model development and evaluation more difficult [91].

Model behaviour itself introduces additional challenges. While different architectures may achieve similar aggregate performance metrics, they often rely on divergent features for decision-making, raising concerns regarding robustness and interpretability [92]. Furthermore, performance may decline as classification tasks become more complex or involve multiple disease categories [93]. This may make clinical implementation of multi-disease screening tools more difficult.

A third consideration for deployment is financial integration: AI systems in other specialties have paved the road for a two-channel payment model in the USA: use of a “New Technology Add-On Payment” for the minority cases taken on in the inpatient setting, and on the outpatient side, migration from a CPT category 3 code to a CPT category 1 code for reimbursement as an AI diagnostic system matures [94].

Finally, the niche that these models will occupy is also an important factor in understanding the route to adoption: Fields with limited specialist availability or challenging diagnosis and treatment decision-making, such as RoP [89], may be among the earliest to see clinical adoption for the purposes of triage, referral and decision support. Overall, if AI systems are to achieve clinical implementation, ongoing research must focus not only on the efficacy of these systems at current diagnosis, but also on the financial and quality of care improvements that AI systems bring to ophthalmology.

### Health Economics

The economic impact of AI-based screening has been most extensively studied in the context of DR, where evidence generally supports cost-effectiveness across diverse healthcare systems [95, 96]. Studies from Singapore, Australia and the United States suggest that AI-based screening can reduce costs while maintaining or improving diagnostic performance, particularly when deployed in semi-autonomous workflows [97–99]. Similarly, AI-assisted screening for RoP has been shown to be cost-effective compared with telemedicine-based approaches [82].

However, economic evidence for other conditions, including AMD and glaucoma, is more variable. In some settings, AI-based screening strategies do not seem to be cost-effective relative to standard care, while in others, modest gains in quality-adjusted life years have been demonstrated [100]. If patients can be assessed for multiple diseases within the same imaging session, the cost-effectiveness of AI-based screening may improve [101]. Importantly, the economic impact of AI varies substantially by healthcare setting. In high-income regions, AI may primarily improve efficiency and mitigate workforce shortages, whereas in low-resource settings it may enable screening programmes that would otherwise be infeasible [6, 102]. Nevertheless, implementation remains constrained by infrastructure requirements, including imaging equipment, internet connectivity and referral systems, as well as upfront development and deployment costs [83, 103].

### Accountability, Privacy and Equity

The integration of AI into ophthalmology raises important ethical challenges related to accountability, privacy and equity. The “black box” nature of many DL models limits interpretability, potentially reducing clinician trust and complicating communication with patients [87, 104]. Although explainability methods can provide insight into model behaviour, they do not fully resolve concerns regarding reliability or accountability.

Determining responsibility in cases of diagnostic error remains an unresolved issue. Existing legal frameworks are based on human decision-making, whereas AI-assisted care introduces shared responsibility between clinicians, developers and healthcare systems. Proposed models range from manufacturer liability to clinician oversight or hybrid approaches, but consensus has yet to be established [87, 105]. Privacy and data security are also critical concerns, particularly given the reliance on large-scale imaging datasets. Retinal images can contain biometric information that may enable patient identification and aggregation with clinical data increases the risk of re-identification [106]. Robust data governance frameworks that include de-identification, data use agreements and cybersecurity protections are essential to mitigate these risks [87].

Bias represents a major challenge for the equitable deployment of models. Models trained on non-representative datasets may perpetuate or exacerbate existing disparities in healthcare delivery [87]. Empirical studies have demonstrated differential performance across demographic subgroups, particularly for early or mild disease [43]. Addressing these issues will require improved dataset diversity, subgroup-specific validation and incorporation of fairness-aware model development strategies [105].

### Regulatory Issues

Regulatory frameworks for AI in healthcare are evolving rapidly but remain heterogeneous across regions. The landmark FDA de novo authorisation of LumineticsCore (formerly IDx-DR) in 2018 established the regulatory pathway for autonomous AI systems in ophthalmology in the USA [19, 107]. Overall, the FDA has authorised ~1000 AI-enabled medical devices, most through the 510(k) pathway, which relies on demonstrating equivalence to existing devices rather than independent clinical validation [108–110]. This approach has enabled rapid market entry but raises concerns regarding the adequacy of evidence supporting safety and effectiveness [108, 111, 112]. In Europe, AI regulation is governed by both the Medical Device Regulation (MDR) and the recently introduced AI Act, each with its own regulatory requirements [113, 114]. In China, regulation is supervised by the National Medical Products Administration (NMPA) and involves a stringent multi-step, comprehensive risk management strategy spanning the entire product’s lifecycle, with the regulatory process being based on the type of algorithm being utilised [114]. Regardless of framework, a model’s regulatory designation depends on its level of autonomy and potential risk, with more autonomous models being placed in higher regulatory risk tiers, such as an FDA Class II De Novo or European MDR frameworks’ Class III. While more structurally burdensome to clear, these higher-risk designations provide the regulatory structure necessary for more autonomous and transformative AI systems to safely alter existing clinical workflows.

Several challenges persist across these regulatory paradigms. Current frameworks are primarily designed for static algorithms and may not address continuously learning or adaptive systems adequately. In addition, the broad and evolving capabilities of foundation models and generative AI systems challenge traditional definitions of “intended use” [112]. Relatively few AI systems have been evaluated in prospective, interventional clinical trials, limiting the strength of evidence supporting real-world use.

## Emerging and Future Research

Over the past few years, AI in ophthalmology has begun to transition from AI/DL models trained for specific disease detection or decision-support tasks that rely on a single type of input, towards more broadly applicable systems built on foundation models and LLMs. The introduction of foundation models (e.g., RETFound) demonstrated strong performance across multiple disease detection tasks with the promise of requiring substantially fewer labelled examples than conventional models. This set off a flurry of research activity focusing on evaluating these models for use in various contexts, including primary care, community screening programmes and tele-health workflows [115–118]. Evidence also suggests that models trained using eye imaging may be able to detect extraocular and systemic disease [17, 116]. These approaches may result in more generalisable models to detect both ocular and systemic diseases across datasets, devices and populations, addressing major barriers to clinical adoption faced by earlier AI/DL systems.

### Future Research

Another focus of current work is the use of multimodal LLMs and vision language models (VLMs) that integrate imaging, clinical measurements and free-text clinical descriptions and notes into their predictive process. Recent work shows that LLMs/VLMs adapted for medical domains (e.g., Med-Gemini [119]) can support disease detection by generating imaging reports, calculating risk predictions and providing clinical decision support [117]. Models such as EyeCLIP are trained on large, multimodal ophthalmic imaging datasets along with clinical text. They have demonstrated strong performance across diverse disease detection tasks including rare diseases [120]. A strength of these systems is that in addition to disease detection, they can also help improve explainability by generating a description of the basis for their predictions. Other groups are developing integrated systems that combine an LLM with task-specific foundation models aimed at diagnosis and triage in primary care settings. In one case (Meta-EyeFM [121]), a system achieved an AUC of 0.91 for multi-disease ((DR, AMD, myopic macular degeneration, glaucoma and cataracts) detection, outperforming both general-purpose LLMs and junior clinicians.

This type of architecture (an LLM controller paired with task-specific models) represents a form of agentic AI, where coordinated model components collaborate to perform clinically meaningful tasks. Agentic AI is a system that autonomously coordinates multiple specialised models, tools and processing steps to perform complex tasks such as disease detection, triage and report generation. Rather than a single monolithic predictive model, these systems use LLMs as “controllers” that dynamically route tasks (e.g., image interpretation, risk prediction, report generation) to appropriate sub-units and can iteratively refine outputs. Promising early results (e.g., Meta-EyeFM) suggest that agentic AI systems capable of real-world disease detection across heterogeneous data and settings will see increased interest, with the goal of deployment in clinical platforms.

Beyond ophthalmology, general-purpose AI technologies are increasingly shaping clinical practice across medicine. For example, LLM-based clinical decision support tools such as Open Evidence provide real-time, evidence-based information at the point of care across specialties [122, 123]. Ambient AI documentation tools have also demonstrated significant reductions in Electronic Health Records (EHR) documentation time and clinician task load in multisite evaluations spanning primary care, surgery and other disciplines [124, 125]. EHR-integrated LLM features, such as AI-generated draft replies to patient portal messages, have been deployed across health systems with early evidence of improved efficiency and reduced inbox burden [126, 127]. These assistive tools may reduce administrative burden while introducing comparatively low clinical risk. However, many still lack prospective validation [128].

## Conclusions

Over the past decade, AI has rapidly advanced as a tool for ophthalmic disease detection. It has progressed from largely relying on traditional ML and statistical approaches to DL systems and, more recently, to foundation models and LLM-based approaches built using state-of-the-art AI approaches. Across multiple conditions, AI-based detection systems have demonstrated high diagnostic performance, often comparable to or exceeding expert human clinicians. The first FDA approval for autonomous AI-based disease detection was for DR detection. These successes have established ophthalmology as a leading field in medicine for AI development.

An important evolution in the field has been the shift from relatively limited, task- and modality-specific models toward more generalisable and clinically applicable systems. Traditional ML and early DL approaches could have limited utility because they were typically trained to detect a single disease using a single modality. In contrast, foundation models are trained on large-scale datasets using self-supervised learning with the specific goal of producing generalisable models that can be adapted for a variety of ophthalmic tasks. They have demonstrated the ability to adapt to multiple disease detection tasks even in cases where only limited labelled data is available. Emerging LLM and agentic approaches offer the potential to incorporate multimodal data better, provide clinical decision support in addition to disease detection and, using agentic approaches, integrate with existing models and tools within clinical workflows.

However, as highlighted in this article, the translation of these technologies into routine clinical practice remains limited and several barriers still need to be addressed to increase clinical adoption. These barriers span from technical and clinical issues to ethical and regulatory concerns, as well as economic and cost considerations. One fundamental technical concern is ensuring that the high model performance observed in research settings is preserved in real-world clinical settings. Variability in imaging quality, data heterogeneity, differences across devices and settings and demographic underrepresentation can all impact model performance. Issues related to model transparency, bias and data privacy are also relevant to these concerns and directly influence model performance and impact on patient care. Each of these considerations needs to be incorporated into additional prospective evaluations in diverse clinical settings to ensure models are achieving acceptable performance in clinical deployment.

Economic and regulatory considerations also impact the ability to translate these tools into clinical care. AI-based disease detection has demonstrated consistent cost-effectiveness in DR screening, particularly in settings with limited access to specialist care. However, evidence for other diseases is more variable and the value of AI depends on the healthcare context. In low-resource settings, AI may enable screening programmes that would otherwise be infeasible, while in higher-resource environments, benefits may be more closely tied to workflow efficiency and capacity expansion. Regulatory frameworks have begun to accommodate AI-based diagnostic tools, but remain primarily designed for static, single-purpose systems and may not fully address the complexities of newer, dynamic models, especially LLM or agentic-focused tools.

In conclusion, AI-based disease detection in ophthalmology has achieved remarkable technical progress, but its clinical impact will depend on addressing key translational barriers. Future work should prioritise prospective evaluation in real-world settings, addressing model transparency and bias and alignment with clinical and regulatory requirements. With continued progress, AI has the potential to transform the practice of ophthalmology and preserve vision for patients.

## Data Availability

No datasets were generated or analysed during the current study.
